# A mechanistic understanding of the effects of polyethylene terephthalate nanoplastics in the zebrafish (*Danio rerio*) embryo

**DOI:** 10.1038/s41598-023-28712-y

**Published:** 2023-02-02

**Authors:** Narmin Bashirova, David Poppitz, Nils Klüver, Stefan Scholz, Jörg Matysik, A. Alia

**Affiliations:** 1 Institute for Medical Physics and Biophysics, Leipzig University, Leipzig, Germany; 2 Institute for Analytical Chemistry, Leipzig University, Leipzig, Germany; 3 Institute of Chemical Technology, Leipzig University, Leipzig, Germany; 4 Department of Bioanalytical Ecotoxicology, Helmholtz Centre for Environmental Research-UFZ, Leipzig, Germany; 5 Leiden Institute of Chemistry, Leiden University, Leiden, The Netherlands

**Keywords:** Environmental impact, Metabolomics

## Abstract

Plastic pollution, especially by nanoplastics (NPs), has become an emerging topic due to the widespread existence and accumulation in the environment. The research on bioaccumulation and toxicity mechanism of NPs from polyethylene terephthalate (PET), which is widely used for packaging material, have been poorly investigated. Herein, we report the first use of high-resolution magic-angle spinning (HRMAS) NMR based metabolomics in combination with toxicity assay and behavioural end points to get *systems-level* understanding of toxicity mechanism of PET NPs in intact zebrafish embryos. PET NPs exhibited significant alterations on hatching and survival rate. Accumulation of PET NPs in larvae were observed in liver, intestine, and kidney, which coincide with localization of reactive oxygen species in these areas. HRMAS NMR data reveal that PET NPs cause: (1) significant alteration of metabolites related to targeting of the liver and pathways associated with detoxification and oxidative stress; (2) impairment of mitochondrial membrane integrity as reflected by elevated levels of polar head groups of phospholipids; (3) cellular bioenergetics as evidenced by changes in numerous metabolites associated with interrelated pathways of energy metabolism. Taken together, this work provides for the first time a comprehensive system level understanding of toxicity mechanism of PET NPs exposure in intact larvae.

## Introduction

Global plastics production and application are increasing due to their versatility and low costs. However, increasing use of plastic around the world becoming a threat to almost all ecoysystem^1–4^. Plastic debris has been identified in soils, lakes, sediments, oceans, air and even in places with rare human activities^5–9^. It has been estimated that each year 4.8 to 12.7 million tons of plastic waste enter the ocean^10^. One of the major concerns related to plastic pollution is the presence of micro-(MPs) (1 μm–5 mm), and nanoplastics (NPs) (< 1 μm) in the marine ecosystem^11–15^. Various physical, thermochemical, and biological methods are being used for removal of MPs/NPs from various matrices^16,17^. Due to their small particle size, removing such plastic particles through wastewater treatment is still challenging. Consequently, a high amount of microplastic particles entering the marine environment^14^. Several studies are conducted on the transfer mechanism through which NPs are ingested or accumulated in organisms including marine mammals, turtles, fish, birds, and human^18–21^. These NPs enter the organisms via many different routes (e.g., dermal, gastrointestinal, inhalation) and cause hazardous effects, including behavioral variations, fertility changes, weakened immune responses, liver damage, and at high concentration, lethal damage^22–26^. As nanoplastics are essentially inert, their toxicity might be caused by the particulate nature (which is often altered in the biological system due to adsorption with other biomolecules) or by additives or other small organic molecules that leach out after uptake and are hazardous to human health^25,27^. In past years, most toxicological studies have focused on polystyrene nanoparticles (PS NPs)^28–33^. Studies have been carried out using human cell culture or fish larvae to address the toxicological impact of PS NPs and to understand the metabolic pathways affected at cellular and molecular level^28–34^. It has been revealed that PS NPs enter the cell membrane, accumulate in the cell and cause inflammatory response and oxidative damage. However, there are still many gaps in our knowledge on toxicity mechanism and more specifically a comprehensive system level picture of the metabolic pathways affected by nanoplastics.

Polyethylene terephthalate (PET) is the one of most used polymers for practical container (container for food, drinks, plastic bags etc.) that stands out for its transparency, flexibility, and innocuity^35^. It is also used for textile, parts of automotives and electronics^35,36^. Although, several studies addressing the toxicity of nanoplastics focused on presence of other polymer, e.g., polystyrene (PS) nanoparticles^28–32^, the toxicity associated with PET and toxicity mechanisms, remain largely unstudied. PET particles have been found in groundwater, drinking water, soils and sediments and in the air^30,31,35,37^. The hazardous effects of PET in the form of nanoparticle (PET NPs) in various marine organisms such as amphipods, copepods, and fish has been documented^38,39^. PET NPs have raised severe concern regarding potential danger and risk for nature and human well-being^35,37^. Studies on human cell culture showed that PET NPs at higher concentration have inhibitory effect on cell viability^37,40^, and the interaction of PET NPs with different contaminants (Hg^2+^, glyphosate and levofloxacin) can significantly change the cell physiology^41^. Using human lung carcinoma cell culture, Zhang *et al.*^37^ have shown that PET NPs increases reactive oxygen species (ROS) which may affect mitochondrial potential. Whether PET NPs can affect cellular metabolism is not yet systemically studied. A comprehensive system level tracking of the toxicity pathways affected by PET NPs is necessary to understand the toxicity mechanism of PET NPs.

Zebrafish (*Danio rerio*) is used as a model organism in a wide range of developmental toxicological studies^42–47^. Performing developmental toxicity studies using the zebrafish embryo model is advantageous for multiple reasons; (1) to generate a robust sample for downstream applications (transcriptomics, proteomics, and metabolomics) a large number of zebrafish embryos can be exposed in small volumes (e.g., ~ 10 embryos per mL); (2) the pore size of the chorion (0.5–0.7 µm) allows to study a wide range of small molecules^48^; (3) various developmental stages, from fertilized egg to free-swimming larvae can be studied in short time duration (from 1 h up to few days); (4) a growing range of biochemical and molecular technologies including “omics” approaches have been adapted to zebrafish embryos and larvae^42–45^. Among these, several mass spectrometric (MS) and nuclear magnetic resonance (NMR) spectroscopic tools are available for extracts obtained from zebrafish embryos^49–51^. Compared to other techniques high-resolution magic-angle spinning (HRMAS) NMR is a non-destructive technique used to study metabolic profile in intact tissues or organism^43–46^. In earlier studies, HRMAS NMR has been successfully utilized to investigate alteration of metabolic profiles by various toxicants in early life stages, i.e., embryos and larvae, of the zebrafish^43–46^.

The aim of the present study is to assess the potential toxicity of PET NPs on zebrafish embryos and to get comprehensive system level understanding of the metabolic pathways affected by PET NPs. Although zebrafish embryo has been used previously to investigate the toxicity of PET NPs^39^, the current study is among the first to investigate the toxicity mechanism at whole organism level using non-destructive metabolomics approach of HRMAS NMR. Our results show convincing evidence that PET NPs significantly affects liver functioning, induces oxidative stress, affect cellular membrane and energetics.

## Results

### PET nanoparticle characterization

**Figure 1:**
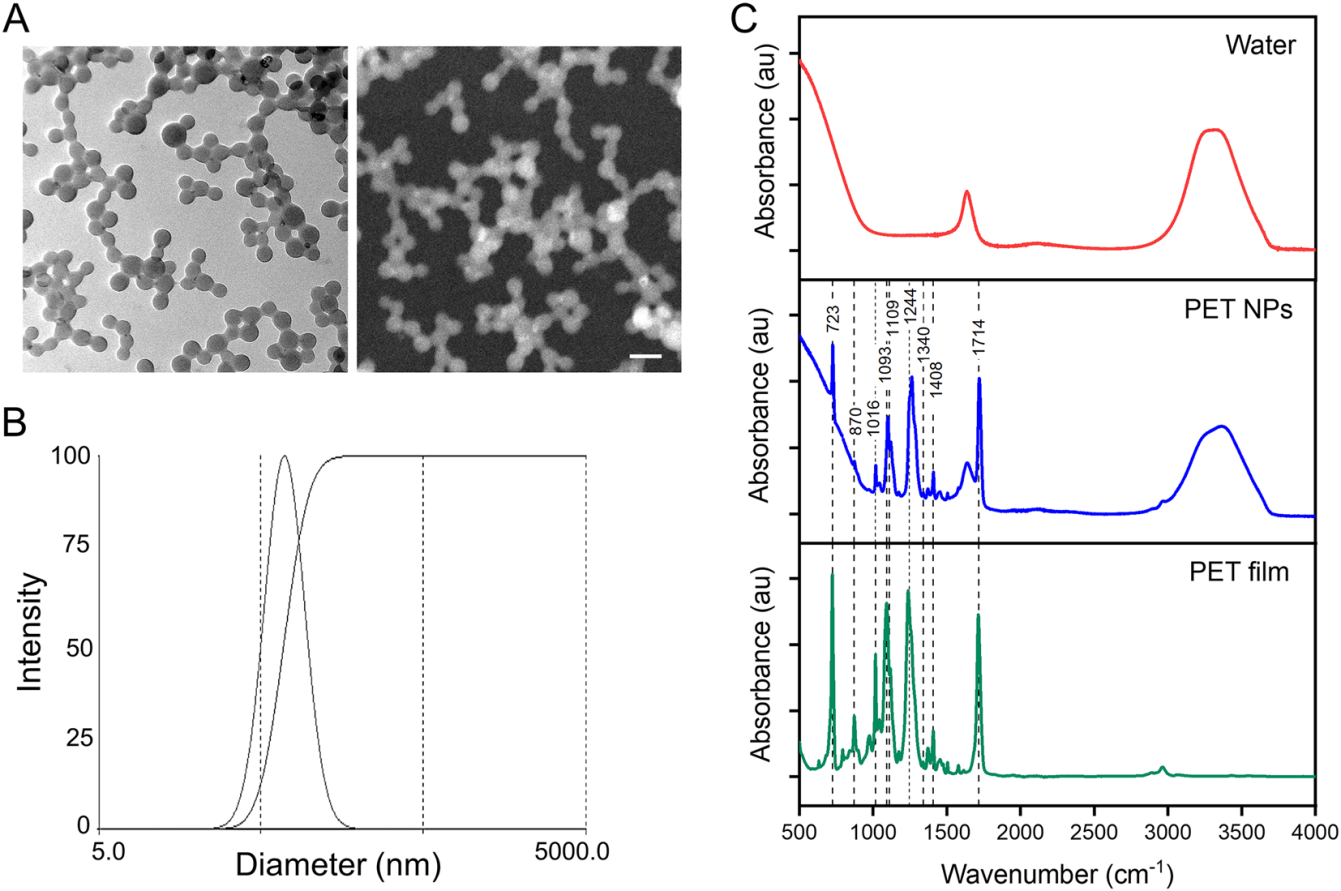
(**A**) Transmission electron microscopy (TEM) image (left) and high-angle annular dark-field scanning transmission electron microscopic (HAADF-STEM) image (right) (scale bar: 100 nm); (**B**) Hydrodynamic diameter distribution of PET NPs determined by dynamic light scattering; (**C**) ATR-FTIR spectra of water, PET NPs suspension and PET film.

The stable PET nanoparticles were successfully prepared as described in method section. The PET NPs suspension remained stable for several weeks without any settling. The transmission electron microscope (TEM) imaging was performed to characterize the morphology of the PET NPs. The TEM imaging shows that PET NPs are composed of small spherical structures (Fig. 1A). In order to investigate details of the distribution/segregation of these elements in and surrounding of nanoparticles material contrast imaging by high-angle annular dark-field scanning transmission electron microscope (HAADF-STEM) was performed. In the HAADF-STEM imaging material contrast for heavier elements is represented by higher contrast (Fig. 1A). Thereby, out of the images the nanoparticles can be identified having an equal material distribution**.** These results show that PET starting material used was rather pure and does not contain any contamination of other elements and metal ions.

The hydrodynamic size distribution of PET NPs was characterized by dynamic light scattering (DLS). The hydrodynamic diameter was found to be 70 ± 5 nm. Their hydrodynamic diameters were consistent when dispersed either in distilled water (DW) or in egg water (ISO water) used for the zebrafish embryo experiments (Fig. 1B). Zeta potential value of the NPs was also determined and indicates that these NPs were negatively charged with values of − 20 ± 5 mV. The polydispersity index (PDI 0.08) value showed nanoparticles were homogenous and had low aggregation property, and they remained stable for several weeks without any settling.

Further we determined the FTIR-ATR absorption spectra of PET NPs in aqueous solution and in amorphous PET film (Fig. 1C). The marked bands attributed to PET at strong carbonyl stretching band at 1714 cm^-1^ (C=O stretching vibration), 1408 cm^−1^ (aromatic skeletal stretching vibrations)^52^, a doublet near 1100 cm^−1^ (C–O stretching), the strong absorptions at 1244 cm^−1^ (C(=O)O stretching, ring-ester C–C stretching, and C–O in plane bending) and 723 and 870 cm^−1^ (bending group (out of plane) of benzene ring). The FTIR analysis showed that no change occurred between the chemical composition of PET NPs and PET film.

### The effects of PET NPs on zebrafish embryo

**Figure 2:**
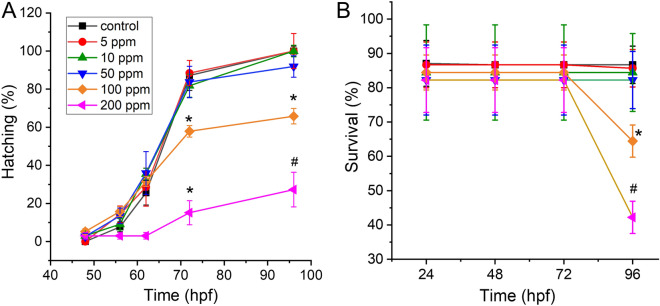
Effect of PET NPs on hatching (**A**) and survival rate (**B**) of zebrafish embryos. Embryos at 6 hpf were exposed to different concentrations (i.e., 0, 5, 10, 50, 100 and 200 ppm) of PET NPs for 24, 48, 72 and 96 h. Values shown are the mean ± standard deviation (n = 30 embryos per group). For statistical analysis, one-way ANOVA was performed using OriginPro v.8 (OriginLab, Northampton, MA, USA). The statistically significant differences in hatching and survival rate between PET NPs treated and control group obtained by ANOVA analysis are indicated by *p < 0.05 and # *p* < 0.01 as compared to untreated controls.

**Figure 3:**
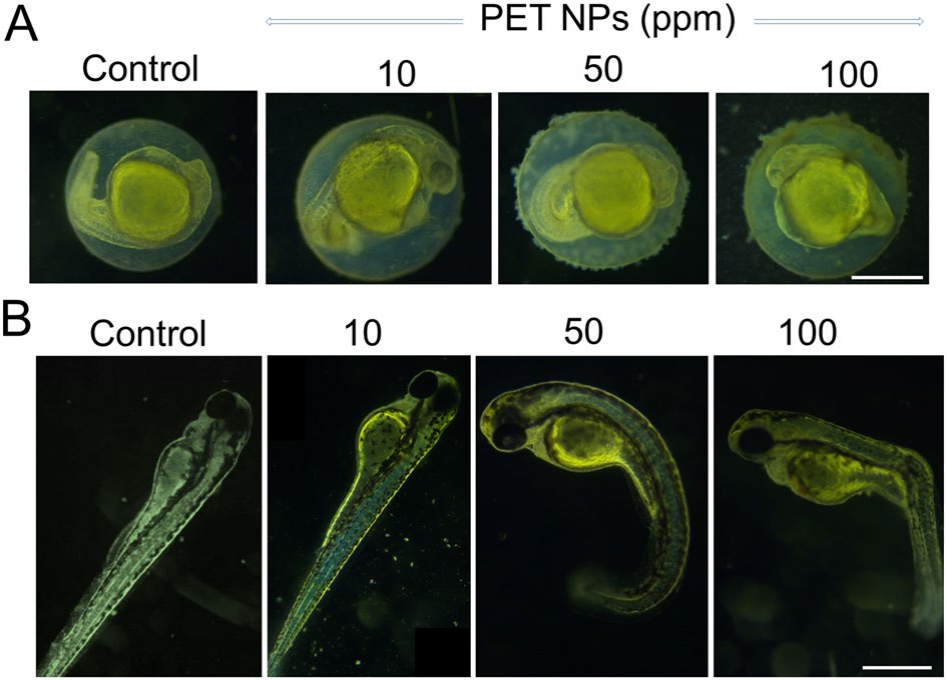
Representative images showing the effect of PET NPs on early stages of zebrafish embryo development. Embryos (6 hpf) were treated with different concentrations of PET NPs (0, 10, 50 and 100 ppm) for 24 h (**A**) and 72 h (**B**). After 72 h exposure to 100 ppm PET NPs, approximately 6–8% of the embryos show deformities including malformation of lateral curvature in spine similar to scoliosis, bending of the tail and bending of the body after treatment with PET. Scale bar: 0.5 mm in (**A**) and 1 mm in (**B**).

To evaluate biological effect of PET NPs on zebrafish embryo development, hatching rate, survival rate, and morphological deformities were recorded over a range of PET NPs concentrations (0, 5, 10, 50, 100 and 200 ppm) by exposing embryos from 6 h post fertilization (hpf) to 96 hpf. Compared with the control group, embryos exposed to concentration of 5–50 ppm of PET NPs did not show significant effect on hatching rate. However, embryo-hatching rate was significantly reduced at and above 100 ppm PET NPs. Only two thirds of the population completed hatching at 96 hpf after exposure of embryos to 100 ppm PET NPs (*p* < 0.05) while only one third hatched at 200 ppm PET NPs (*p* < 0.005) treatment (Fig. 2A). In addition, we evaluated dose dependent effect of PET NPs on embryo survival (Fig. 2B). As shown in Fig. 2B, embryos exposed to lower concentrations of PET NPs (e.g., ≤ 50 ppm) did not induced any effect on survival rate as measured until 96 hpf. However, at 100 ppm and 200 ppm concentration of PET NPs, the survival rate was declined to 64% and 42%, respectively. Figure 3A shows representative images of the 24 hpf embryos exposed to different concentration of PET NPs. Apart from delay in hatching as mentioned above, the effect of PET NPs treatment for 24 h at early developmental stage did not show any significant effect of their growth. Interestingly at higher concentrations (e.g., at ≥ 50 ppm), PET NPs showed aggregation on the surface of chorion. The lower hatching rate at higher concentration of PET NPs as seen in Fig. 3A, may be due to these aggregation of PET NPs on the surface of chorion.

Since we had observed concentration-dependent toxicity on hatching and survival rate we further investigated if survived zebrafish embryos have any deformities at various PET NPs exposed zebrafish embryos. Compared with control, about 6–8% of the survived embryos exposed to PET NPs at concentrations of 100 ppm for 96 h exhibited some effects on their morphology (Fig. 3B). The most commonly observed deformities were malformation of lateral curvature in spine similar to scoliosis (e.g., 100 ppm).

In order to evaluate the effect of PET NPs at late developmental stage of zebrafish embryo, we treated the fully hatched control zebrafish embryos (72 hpf) with various concentrations of PET NPs (0, 5, 10, 50, 100 and 200 ppm) for 24 h. No significant mortality rate was observed in larvae from all exposure groups (after 24 h, 48 h treatment with PET NPs) relative to untreated control group (data not shown).

Further we analyzed functional endpoints in the zebrafish embryos such as locomotor activity and heart rates in the different exposure groups. Zebrafish embryos exposed from 24 to 96 hpf (72 h exposure duration) with 100 ppm PET NPs resulted in a reduced locomotor activity (hypoactivity) in the dark phase compared to controls, whereas lower PET concentrations did show a similar light/dark activity as the controls (see Supplementary Fig. S1A, B). However, exposures up to 100 ppm PET NPs did not affect the heart rate in zebrafish embryos (see Supplementary Fig. S1C).

### Uptake and biodistribution of PET NPs

**Figure 4:**
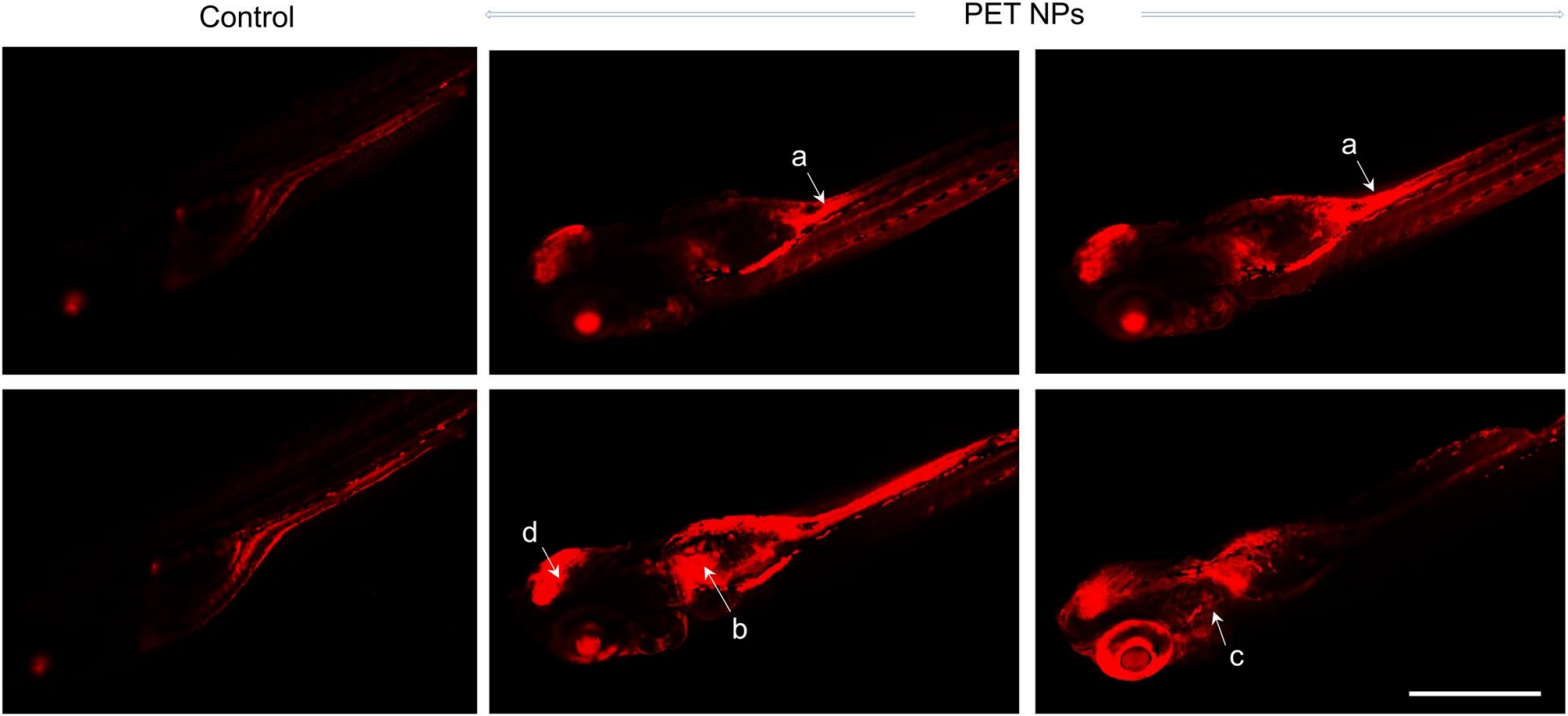
Representative Fluorescent confocal images (successive slices) showing the distribution of PET-NR nanoparticles in the body of zebrafish embryos (96 hpf) that were exposed to 100 ppm PET-NR nanoparticles for 24 h as compared to embryos treated with only NR (control). As can be noticed, nanoparticles are accumulated at various locations including intestine (a), pronephric duct/kidney (b), liver (c) and brain. Images were acquired using inverted laser-scanning confocal microscope (Leica DMi8 / TL LED, Leica Microsystems CMS GmbH). Scale bar: 1 mm.

To more specifically evaluate the uptake and biodistribution of the PET NPs, we labelled PET NPs with Nile red (PET-NR). Zebrafish embryos (96 hpf) were exposed to 100 ppm PET NPs-NR for 24 h. Confocal laser scanning microscope (CLSM) images are shown Fig. 4. CLSM images showed the intercellular fluorescence of NR labeled PET NPs in various locations including intestine, kidney, liver and brain. At 96 hpf, the gastrointestinal (GI) tract is fully functional (due to the larva resorbed the yolk sac), therefore, the accumulation of PET NPs was very prominent in the intestine and liver^13^. These results indicated that PET nanoparticles, were taken up by zebrafish larvae and they can readily accumulate in most tissue of the embryonic bodies.

### NMR-based metabolic profiles of zebrafish exposed to PET NPs

**Figure 5:**
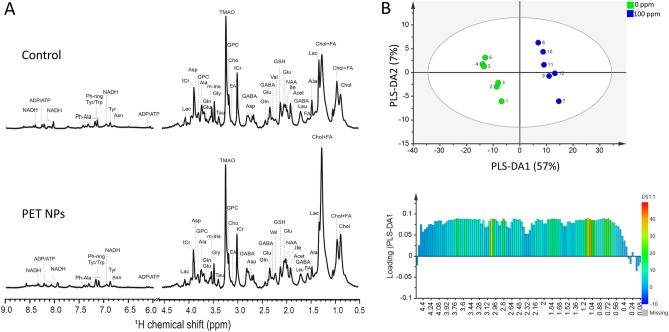
(**A**) Representative high-resolution magic angle spin (HRMAS) NMR spectra of control and PET NPs (100 ppm) exposed zebrafish embryos. 72 hpf zebrafish embryos were treated with PET NPs for 24 h. Red arrows indicate an increase and decrease of metabolites. Abbreviations: Ala = alanine; Asn = aspargine; Asp = Aspartate; ADP = adenosinediphosphate; ATP = adenosinetriphosphate; Cho = choline; Chol = cholesterol; EA = Ethanolamine; FA = fatty acid; GABA = γ-aminobutyric acid; Gln = glutamine; Glu = glutamate; Gly = glycine; GPC = glycerophosphocholine; GSH = glutathione; Lac = lactate; m-Ins = myo-inositol; NAA = N-acetylaspartate; NADH/NAD +  = reduced/oxidized nicotinamide adenine dinucleotide; Phe = phenylalanine; Tau = Taurine; tCr = total creatin; Trp = tryptophan; Tyr = tyrosine; TMAO = trimethylamine N-oxide. (**B**) Multivariate analysis of the HRMAS NMR spectra (n = 6 per group) using orthogonal partial least square-discriminant analysis (PLS-DA) modelling (R2 = 0.998, Q2 = 0.962). (upper) Scores plots (PLS-DA1 vs PLS-DA2). The score plot explains 64% of total variance of control clustering in the positive PLS-DA2 scores, and PET in the negative PLS-DA2 scores. (lower) Loading plots of PLS-DA1 for all buckets containing assigned peaks.

Metabolic profiles of zebrafish embryo controls and embryos treated with 100 ppm PET NPs for 24 h (72–96 hpf) were obtained by 1D ^1^H HRMAS NMR (Fig. 5A). Several metabolites were identified by comparison of the 1D NMR chemical shifts to the Human Metabolome Database (HMDB), along with 2D NMR technique (i.e., ^1^H-^1^H COSY) (see Supplementary Fig. S2).

The 1D HRMAS NMR spectra were further analysed by multivariant analysis. PLS-DA model was used to discriminate the metabolite distribution of PET NPs treated groups from controls. Clear group discrimination was observed in PLS-DA score plot (Fig. 5B). The pattern recognition result showed that control groups clearly separated from treated group (100 ppm). Control group embryos were clustered towards the positive PLS-DA1 scale while PET treated embryos were clustered towards the negative PLS-DA1 scale. These results suggest that treated group has altered metabolic profiles as compared to control group. Metabolites assigned to the corresponding buckets to determine which variables are mainly responsible for the separation of two groups and the load values of the PLS-DA1 are given in Fig. 5B.

**Figure 6:**
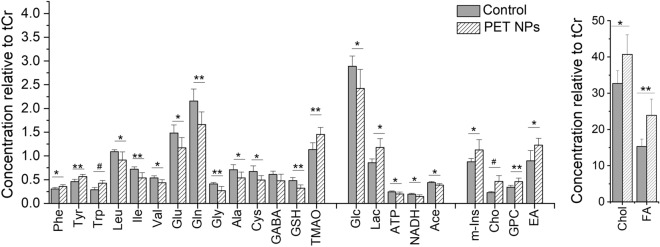
Effect of PET NPs treatment on the metabolic profile of intact zebrafish embryos. Zebrafish embryos (72 hpf) were exposed to 100 ppm PET NPs for 24 h. Concentrations of metabolites relative to total creatine (tCr) are shown. For statistical analysis, one-way ANOVA with a Tukey post-hoc correction for multiple comparisons were performed using OriginPro v. 8 (Northampton, MA, USA). Values shown are the mean ± standard deviation (n = 6). The statistically significant differences in metabolites between PET NPs treated and control group obtained by ANOVA analysis are indicated by # *p* < 0.001, ** *p* < 0.01 and * *p* < 0.05. Abbreviations: Phe = phenylalanine; Trp = tryptophan; Tyr = tyrosine; Leu = leucine, Ile = isoleucine; Val = valine; Glu = glutamate; Gln = glutamine; Gly = glycine; Ala = alanine; Cys = cysteine; GABA = g-aminobutyric acid; GSH = glutathione; TMAO = trimethylamine N-oxide; Glc = glucose; Lac = lactate; ATP = adenosine triphosphate; NADH = nicotinamide adenine dinucleotide; m-Ins = myo-inositol; Cho = choline; GPC = glycerophosphocholine; Chol = cholesterol; FA = fatty acids.

The quantitative analysis of the metabolites in HRMAS NMR spectra revealed significant decreases in the levels of acetate (Acet), glucose (Glc), alanine (Ala), leucine (Leu), isoleucine (Ile), valine (Val), glutamate (Glu), glutamine (Gln), cysteine (Cys), glycine (Gly) and glutathione (GSH) (Fig. 6). Decrease was additionally observed for ATP and NADH. Significant increase on the other hand was observed in the levels of lactate (Lac), choline (Cho), glycerophosphorylcholine (GPC) and ethanolamine (EA), tryptophan (Trp), phenylalanine (Phe) and tyrosine (Tyr). Increase in free fatty acids (FA), cholesterol (Chol) were also observed in exposure group compared to controls. ANOVA test results clearly show statistically significant changes in metabolites of zebrafish embryos exposed to PET compared to controls are shown in Table S1.

### Oxidative stress in PET NPs exposed zebrafish embryos

**Figure 7:**
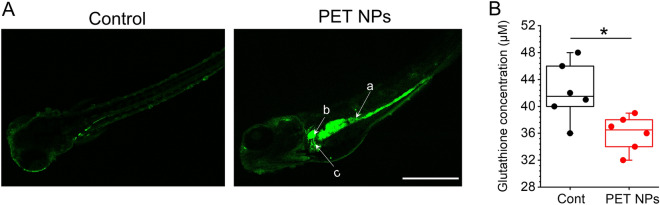
(**A**) Localization of reactive oxygen species (ROS) production in zebrafish embryos exposed to PET NPs (100 ppm) as compared to control embryos. Embryos (96 hpf which were already treated with PET for 24 h) were incubated for 60 min in CM-H2DCFA (10 μM) in rearing medium. Fluorescence detected in different regions of PET NPs treated embryos including (a) Intestine), (b) gall bladder and (c) liver. Scale bar: 1 mm. (**B**) Glutathione levels in extracts of zebrafish embryos (72 hpf) exposed to PET NPs (100 ppm for 24 h) as compared to control (Cont) embryos. Glutathione (GSH) levels were analysed by using GSH assay kit from Sigma-Aldrich. Significant reduction of GSH (*p < 0.05; n = 6) in PET NPs-treated embryo is clearly observed.

In vivo visualization of reactive oxygen species (ROS) generation by PET NPs (100 ppm) was examined using a previously developed method based on the fluorescent probe, chloromethyl-2',7'-dihydrodichlorofluorescein diacetate . Significantly higher levels of ROS were observed in embryos treated with PET NPs, compared to controls, and specifically in the intestine, liver, and kidney regions (Fig. 7A). This observation is consistent with specific uptake and accumulation of PET NPs in these regions. The observed increase in ROS signifies an oxidative stress induced by exposure of PET NPs to zebrafish embryos. Consistent with oxidative stress, zebrafish embryos (at 96 hpf) exposed to PET NPs showed a significantly lower level of glutathione which is a key cellular antioxidant molecule that protect against oxidative stress (Fig. 7B).

## Discussion

PET is one of the most widely manufactured plastics today. PET NPs are an emerging, yet poorly understood environmental contaminant of global concern. Despite the potential risk and adverse effects of other nanoplastics such as polystyrene (PS NPs) are relatively well investigated, the potential toxic effects of PET NPs in biological organism and the underlying mechanisms of toxicity remained largely unstudied. In this work we have systematically studied the potential physiological and metabolic effects associated with exposure to PET NPs. Using non-destructive HR MAS based metabolomics, we provide a comprehensive system level understanding of the metabolic pathways affected by PET NPS in zebrafish embryo model.

At lower concentration (e.g., at ≥ 50 ppm), PET NPs did not inflict acute toxicity in terms of hatching rate or survival of the embryos; however, exposure to higher concentration of PET NPs (100 ppm) influenced the hatching success as well as embryo survival. The lower hatching rate at higher concentration of PET NPs, may be due to the aggregation of PET NPs at the surface of chorion as observed in our study. The pore canals in the chorion are about 0.5–0.7 µm in diameter^48^. It is likely that a large PET NPs aggregate, at the surface of the chorion, might have blocked the pore canals the of chorion and interfered with the transport of oxygen/carbon dioxide and nutrients (Fig. 3A). Furthermore, the adsorption of PET NPs at the surface of chorion might block the release of secretory chorinase by hatching glands that is responsible for initiating hatching gene-expressions^53^. Similar effect of compromised barrier function of zebrafish embryonic chorions against other nanoplastic such as polystyrene NPs and its impact on hatching embryo development has been reported earlier^54^. In another study using BSA-capped PET NPs, a hatching inhibition effect of zebrafish embryos has been observed^39^. In addition to the effect on hatching and survival, we observed that exposure of high dose (100 ppm) of PET NPs at late developmental stage of zebrafish embryo can cause behavioral changes which was observed in term of reduced locomotor activity (hypoactivity) in the dark phase compared to controls.

To explore the uptake and distribution of PET NPs we utilized the fluorescent-labelled NR labeled PET NPs, which show that PET NPs get localized at various places including intestine, liver, and kidneys (Fig. 4). These results are in agreement with previous studies, which have shown that gastrointestinal system is a major target for both direct (e.g., intentional ingestion) or indirect (e.g., nanoparticles from food containers) contamination by nanoplastics^39,55–57^. As the nanoplastics have been shown to pass across the gut epithelium of anterior intestine, their distribution can be extended to other parts of the body, thus has potential for systemic exposure^57^. To assess the system level toxicity mechanism of PET NPs, it would be necessary to understand metabolic pathways affected by PET NPs exposure. For this we utilized state-of-the-art non-destructive HRMAS NMR based metabolomics on intact embryos exposed to PET NPs.

HRMAS NMR based metabolic profile showed that numerous metabolites associated with interrelated pathways of carbohydrate, amino acid and lipid catabolism were altered in PET NPs exposed embryos (Figs. 5, 6). Interestingly, we observed a significant decrease in Ile, Val, and Leu. These are branched chain amino acids (BCAA) which are known to promote metabolism of fatty acids and prevent fat accumulation in live^58^. Alterations of these metabolites indicated that exposure to PET NPs induced a disruption of lipid metabolism in liver, which is in line with the observed accumulation of PET NPs in the liver and observed increase in lipid metabolites. It has been demonstrated earlier that other type of nanoplastics e.g., PS NPs can be efficiently absorbed by zebrafish liver cells and affect expression of genes involved in lipid metabolism^33,58,59^. It has also been proposed that PS NPs might affect the balance of gut microbes, which are important for lipid metabolism in the liver^59^. Remarkably, we also observed significant increase in aromatic amino acids (AAA; Phe, Tyr, Trp) in PET NPs exposed embryos (Fig. 6; Supplementary Table S2). It is well known that during compromised liver functioning, the ratio of aromatic amino to branched-chain amino acids increase^45,47,60^. Thus, an increase in ratio of AAA to BCAA in PET NPs treated embryos further indicates that PET NPs causes liver impairment. Furthermore, effect on liver is indicted by an increase in TMAO, which has been proposed in many studies as a potential biomarker of the liver damage^44,46,61^. Targeting of liver by PET NPs would also be consistent with the toxicity observed with PET NPs at later stages of embryo development (Fig. 2), since it has been shown that differentiation of the liver, and significant expression of relevant embryos enzymes (e.g. cytochrome P450) in zebrafish occurs only after at approximately 72 hpf^44^.

Possible targeting of the liver by PET NPs coincides with alterations of antioxidant and detoxification pathways as evidenced by decrease in metabolites directly or indirectly associated with oxidative stress and detoxification pathways. A significant decrease in GSH was measured by HRMAS NMR following PET NPs exposure (compared to controls). The decrease in GSH was further confirmed, in the present study, by ex vivo colorimetric assay (Fig. 7). Furthermore, we see decrease in the levels of Glu, Gly and Cys which are biosynthetic precursors of GSH, and decreases in these metabolites could, therefore, additionally explain the observed GSH decrease. Indeed, it has been well established that reduction in the precursors (i.e., Glu, Gly and Cys) can, in fact, lead to cellular depletions of GSH which in turn intensify oxidative stress and damage^62^. The oxidative stress was confirmed by observing an increased ROS production in various regions including liver in PET NPs exposed zebrafish embryos. In fact, in vivo ROS production in various parts of the embryos coincide well with distribution of PET NPs in these organs (i.e., liver, intestine, kidney, and brain) (Figs. 4, 7). Our results of compromised detoxification pathways with PET NPs are in line with earlier studies, which showed pro-oxidant properties of micro- and nano-sized PS particles leading to decreased levels of reduced GSH, and increased ROS levels and altered antioxidant enzyme activities^12,28,29,63–65^.

A key consequence of increase ROS include oxidative damage to cellular membranes. In this regard,, we observed a significant increase in FA, Chol, Cho, EA and GPC in PET NPs exposed embryos. These metabolites are associated with lipid composition of cell membranes: Cho, GPC, EA and m-Ins form polar head of many phospholipids, and fatty acids form “non-polar tails,” whereas cholesterol has an established role in maintaining cell membrane integrity and fluidity, and can comprise, in fact, as much as 50% of cell membranes^62^. As such, the combined observation of elevated levels of these metabolites by HRMAS may represent either a direct possible interactions of PET NPs with membrane lipids, and subsequent disruption of membranes. or alternatively, a consequence of ROS induced oxidative damage to cellular membrane. Accordingly, disruption of cellular membranes could be postulated to potentiate consequent hydrolysis of phospholipids and consequent release of free Cho, GPC, EA and FA. Increase in these metabolites has, in fact, been shown to be linked with membrane disruption in previous studies involving zebrafish embryos exposed to toxins^43,44,61,62^. Among various cellular membrane, effect of PET NPs on mitochondrial membrane may have severe consequences on energy metabolism. Previous metabolomics studies on in vitro human cell culture exposed to polystyrene NPs have shown that PS NPs affect mitochondrial membrane and affect membrane potential and energy homeostasis^30^. Thus, increase in free phospholipid metabolites due to PET NPs exposure may be associated with damage to mitochondrial membrane.

Another observation of this study is the significant changes in several interrelated metabolites associated with energy metabolism. Among these, is a decrease in Glc and Acet and increase in Lac. Decreases in Glc alongside an increase in Lac would be indicative of anaerobic glycolysis. The decreased Acet may be related to mitochondrial functions that may have been affected by exposure to PET NPs; this further influenced acetyl-CoA metabolism and finally TCA cycle and thus induced the decrease in the level of Glu. Both processes are the main part of energy metabolism. Disruption of energy metabolism is also evidenced by the decreased levels of ATP and NADH. As the large amount of ingested PET NPs have no nutritional value, they might interfere with the food absorption which in turn leads to alteration in energy and lipid metabolism. These results are in line with previous studies on other microplastics which have shown that ingested MPs deplete energy reserves in marine worms, copepods and affect the feeding activity of fish^59,66–68^. Earlier transcriptome studies of liver from mice exposed to PS MPs showed that they inhibit multiple biological processes related to energy metabolism, such as glycolysis, glucose transport, fatty acid synthesis, and oxidation^69^.

Overall, the alterations in metabolites in PET NPs exposed embryos signify that target of PET NPs toxicity includes: (1) organ-specific targeting of liver and associated systems, e.g., kidney and intestine; (2) corresponding effects on antioxidant and detoxification pathways; and (3) affecting cellular membranes and cellular bioenergetics.

## Conclusions

This study provides for the first time an insight into PET NPs-induced toxicity pathways and the underlying toxicity mechanisms in intact zebrafish larvae. In particular, the result identified toxic effect of PET NPs on liver and several relevant metabolic pathways suggesting a multifaceted toxicity of PET NPs including: (1) oxidative stress as indicated by increase ROS and decrease in protective antioxidant GSH and its biosynthetic precursors Glu, Gly and Cys; (2) disruption of mitochondrial membrane integrity and hydrolysis of phospholipids as indicated by elevated levels of free fatty acids and free polar head groups of phospholipids; (3) cellular bioenergetics as evidenced by changes in numerous metabolites (e.g. glucose, lactate, acetate, ATP and NADH) associated with interrelated pathways of energy metabolism. Based on metabolic profiles, and complementary assays, an integrated model of PET NPs induced toxicity is proposed. Our model suggests that PET NPs cause hepatotoxicity which compromises detoxification and antioxidant pathways, leading to mitochondrial membrane dysfunction manifested by cross-talk between pathways of energy metabolism. The present study also demonstrates the considerable potential of HRMAS NMR based metabolomics when coupled to zebrafish model for systems-level assessments of mechanism of toxicity of nanoplastics.

## Materials and methods

### Chemicals

All chemicals were purchased from Sigma-Aldrich (St. Louis, MO, USA) unless otherwise stated.

### PET nanoparticles preparation

PET NPs was prepared based on the previous method with slight modifications^70^. Briefly, 80 mg of amorphous PET (Goodfellow, Germany) was dissolved in 8 ml Hexafluoroisopropanol (1% v/v) at room temperature for 12 h. PET suspension was then added to the buret and the solution was added dropwise to ice cooled deionized (DI) water (100 ml) while constantly stirring. Stirring was continued for another 2 h. In order to remove bigger particles suspension was passed through filters (type 12, Cellulose membrane, 125 mm diameter, Roth, Germany). Subsequently, the organic solvent was removed from solution using a rotatory evaporator (Heidolph Instruments, USA) at elevated temperature and reduced pressure (50 °C, ~ 250 mbar). Next, the nanoparticles were allowed to settle in the cylinder for 2 h and the top 40 mL suspension was collected. Concentration of nanoparticles was determined gravimetrically by drying 2 mL of suspension in a pre-dried polymer pellets (3x) at 50 °C for 24 h and further weighing them to quantify the residual. The prepared solution was stored at room temperature, and it was stable for several weeks without settling.

### Transmission electron microscopy (TEM)

TEM was used for imaging and to evaluation of the size of the PET NPs. Sample preparation was performed by transferring a drop (10 μl) of the PET NPs solution (0.1 mg/mL) on a holey carbon TEM grid, allowed to settle and dried at room temperature. The TEM analysis was performed on a JEM2100Plus (Jeol, Japan). The microscope is equipped with a LaB6 filament and was operated at an acceleration voltage of 200 keV. Elemental analysis was performed by energy dispersive X-ray (EDX) analysis, using a windowless Optima-T-30 detector (EDAX, UK). Furthermore, the images were recorded with a 4 K ultrafast CMOS camera system (TVIPS, Germany) and analyzed by the relating camera software EMMeasure (TVIP, Germany).

### Dynamic light scattering (DLS)

DLS was used for detection of the size of nanoparticles in solution using Zeta Potential & Particle Size Analyzer (ZetaPALS, Brookhaven, US). Samples were allowed to equilibrate at 25 °C in the sample compartment for at least 2 min and measured in acrylic (10 × 10 × 45 mm, Sarstedt, Germany) cuvettes at 25 °C. The hydrodynamic diameter and zeta potential of PET NPs were then assessed.

### Attenuated total reflectance Fourier transform infrared spectroscopy (ATR-FTIR)

ATR-FTIR was performed using Bruker Alpha II FTIR spectrometer (Bruker Optik GmbH, Germany) equipped with a Diamond Crystal ATR accessory. The spectrum of the Diamond crystal was obtained as background. The samples were prepared by adding 20 µL of solution to the ATR crystal. Absorbance spectra were recorded between the range of 4000 and 399 cm^-1^, at a resolution of 2 cm^-1^ and 128 average scans. Data acquisition performed with Opus 7.8 software (Bruker Optik GmbH, Germany) and the acquired data further plotted using OriginPro v.8 (OriginLab, Northampton, MA, USA).

### Zebrafish husbandry and maintenance

Husbandry and experimental procedures for the collection of zebrafish embryos (OBI/WIK strains) were performed at UFZ Helmholtz Centre for Environmental Research (Leipzig, Germany) according to the standard protocol as described^43,44,61^. Briefly, fertilized and normally developed embryos were sorted and selected using a dissecting microscope, rinsed with egg water (ISO water) several times, and kept in a Petri dish (100 mm × 20 mm; Greiner Bio-One) (N ≤ 100 embryos). ISO water for the standard fish embryo test was prepared as described earlier (ISO 7346-3 (1996) [80 mM CaCl_2_·2H_2_O, 20 mM MgSO_4_·7H_2_O, 31 mM NaHCO_3_, 3.1 mM KCl), pH 7.5]. Embryos were kept in an incubator (Binder, Gmbh, Germany) (14 h light: 10 h dark, 28 °C), and examined daily. All procedures were in accordance with the German animal protection standards approved by the Government of Saxony, Landesdirektion Leipzig, Germany (Aktenzeichen 75-9185.64), and guidelines of the European Union, Directive 2010/63/EU which permits the use of zebrafish embryos up to 120 hpf. All reporting of studies involving use of zebrafish embryos follow the Animals in Research: Reporting In Vivo Experiments (ARRIVE) guidelines^71^.

### PET NPs exposure in zebrafish

To evaluate toxicity caused by PET NPs, zebrafish embryos at two different developmental stages were tested. Initially, 6 hpf embryos were exposed to a range of concentration of PET NPs (0, 5, 10, 50, 100 and 200 ppm) in 6 well plates (3 replicates per treatment group, and 10 embryos/replicate). For the second experiment, 72 hpf embryos were used for exposure studies. This stage was chosen because at this stage of the embryo, morphogenesis is completed and oral uptake and excretions in the larvae are functioning^33^. To keep the concentration of PET NPs constant during exposure period, embryo medium renewed every 24 h. The teratogenic and lethal effects per treatment group were visualized using an Olympus CKX41 (Hamburg, Germany) inverted microscope with phase contrast optics, amounted time-lapse recorder and the analysis software. For this, embryos were first rinsed with embryo medium (ISO, 1996) and anesthetized with tricaine solution (i.e., 200 mg/L) during microscopic imaging. Survival and malformation rate was evaluated as the percentage of dead or deformed embryos per total number of embryos in each replicate, at each test group at regular interval until 120 hpf. Relative (i.e., average) percent survival, hatching and development toxicity at each concentration, and observational time point, were compared by analysis of variance (ANOVA) using OriginPro v.8 (OriginLab, Northampton, MA, USA) to determine statistical significance relative to controls. Temperature was maintained at 28 °C during the experiments.

### Locomotor Response (LMR)

To evaluate behavioural response, caused by PET NPs, zebrafish embryos (24 hpf) were exposed to a range of concentration of PET NPs (0, 5, 10, 50, and 200 ppm) for 72 h. Subsequently, the locomotor response was evaluated at 96 hpf using the ZebraBox video tracking system (Viewpoint, Lyon, France). The LMR test provide specificity and sensitivity of a light–dark transition locomotor response (LMR), where zebrafish embryos exhibit weak movement when illuminated by light but exhibit an increase in activity when switched from light to dark. Thus LMR-L/D measures the swimming activity endpoints such as swimming time, swimming distance, swimming speed (calculated from distance and time) and swimming angle under alternating light/dark cycles^72,73^. The movement of embryos recorded for 45 min in a series of light and dark period (15 min equilibration in light, followed by 10 min light phase and a final 20 min dark phase). The temperature was maintained at 28 (± 1) °C during analysis. The locomotor response of zebrafish embryos recorded for all live embryos, including malformed embryos and embryos showing no inflation of the swim bladder. The behavioral response was assessed based on mean distance moved in the first light phase interval (minutes 15–25) and the dark phase interval (minutes 25–45). For the violin plots the moved distance were normalized to the mean moved distance of the control for the respective phases, as follows:

$$normalized \,\,mean\,\, distance\,\, moved= \frac{mean\,\, distance\,\, moved \,\,sample}{mean \,\,distance\,\, moved \,\,control}$$

### Heartbeat analysis

For heartbeat analysis, Images of zebrafish embryos were first obtained using the VAST Bioimager (Union Biometrica, Gees, Belgium) with the on-board camera of 10 µm resolution as described previously^72,73^. Video frames obtained with the VAST Bioimager system were analyzed using an automated image workflow developed using the KNIME Analytics Platform^72^. Briefly, embryos were anesthetized with a tricaine solution (150 mg/L, TRIS 26 mM, pH 7.5). This tricaine concentration was chosen as this concentration do not affect the heart rate frequency within the time frame (2 h) of analysis^74^. Control and PET NPs exposed embryos were transferred to a rectangular 96-well microplate. The zebrafish heart as the region of interest (ROI) is detected by comparing the absolute difference in pixel intensity between two consecutive frames. By using a threshold method and morphological operations, irrelevant areas were removed from the analysis. Then the pixel variance of the ROI in each frame was used to determine the heart frequency using a Fast Fourier transform with the spectrum function included in the base package of R.

### Visualization of PET NPs

For tracking the biodistribution of PET NPs in zebrafish embryo, PET NPs were labelled with Nile Red (NR) based on method described in an earlier study with slight modification^75^. Briefly, stock solution of NR (0.5 mg/ml) was diluted thousand times with embryo medium. The diluted NR solution was mixed with PET NPs (100 ppm) solution in 1:1000 ratio. The resulting suspension was incubated at room temperature and gently stirred (200 rpm) for 24 h in dark. Five randomly selected zebrafish embryos (72 hpf) were exposed to respective working solution for 24 h, in 6-well plates. In order to remove excess of dye, treated embryos were washed three times with embryo medium and transferred to a new working solution without NR for 24 h. Afterwards, embryos at 120 hpf were placed on a borosilicate glass coverslip and anesthetized with tricaine solution (200 mg/L) during fluorescence microscopy. The fluorescence images of stained zebrafish embryos were captured using inverted laser-scanning confocal microscope (Leica DMi8 / TL LED, Leica Microsystems CMS GmbH) with an excitation wavelength of 552 nm, and an emission wavelength of 636 nm, using a Leica HC PL Apo CS2 (10x/0.40 Dry) objective and Leica Application Suite X (LAS X) software package, version 3.1.5, to acquire images.

### Visualization of reactive oxygen species (ROS)

Generation of reactive oxygen species (ROS) in the control and PET NPs treated embryos was visualized in intact zebrafish embryos at 96 hpf using the probe chloromethyl-2′, 7′ dihydrodichlorofuorescein diacetate (CM-H_2_DCFA; Molecular Probes), which is known as nonfluorescent cell-permeative compound. Intracellular esterases cleave the acetate groups such that the non-fluorescent dye 2′, 7′-dichlorofuorescein (DCF) is retained intracellular and oxidized by intracellular ROS and becomes fluorescent. For sample preparation, CM-H_2_DCFA (1 mM solution in 4% DMSO) was added to control and PET NPs treated embryos in embryo medium to a final concentration of 10 μM, and incubated in the dark for 60 min at 28 °C. Subsequently, excess CM-H_2_DCFA was removed by washing embryos 3 times with embryo medium. Subsequently, embryos were placed on a borosilicate glass coverslip and anesthetized with tricaine solution (200 mg/L) during fluorescence imaging. The fluorescence images of stained zebrafish embryos were captured using inverted laser-scanning confocal microscope (Leica DMi8 / TL LED, Leica Microsystems CMS GmbH). The fluorescent product DCF was detected with an excitation wavelength of 485 nm, and an emission wavelength of 530 nm, using a Leica HC PL Apo CS2 (5x/0.15 Dry) objective and Leica Application Suite X (LAS X) software package, version 3.1.5, to acquire images.

### HRMAS NMR analysis

Metabolic profiling of zebrafish embryos exposed to PET NPs (0 ppm and 100 ppm) was carried out by HRMAS NMR using Bruker DMX 600 MHZ NMR spectrometer (Bruker Biospin GmbH, Germany), which was equipped with a 4-mm HRMAS dual ^1^H/^13^C inverse probe with a magic angle gradient. HRMAS NMR measurements were performed as adapted from previous studies^43,44,62^. For NMR analysis, embryos (72 hpf) were exposed to PET NPs (0 ppm and 100 ppm) for 24 in polystyrene Petri dishes (100 mm × 20 mm; Greiner Bio-One) containing 50 mL of test solution. Exposures were performed with 100 embryos per replicate (n = 6) per exposure group and collected (after 24 h, i.e., 96 hpf) from both controls, and pooled PET NPs. In order to obtain quantitative NMR data, additional replicates for each control and exposure groups were generated. Afterwards, treated and control groups were washed (3 × with MilliQ water) to remove any residual from PET nanoparticles, and zebrafish embryos were transferred into 4-mm zirconium oxide rotors (Bruker Biospin GmbH, Germany). As a reference (^1^H chemical shift at 0 ppm) 10 µL of deuterated phosphate buffer (100 mM, pH 7.0) containing 0.1% (w/v) 3-trimetylsilyl-2, 2, 3, 3-tetradeuteropropionic acid (TSP) was added and the rotor immediately transferred to the NMR spectrometer. For all measurement temperature were adjusted to 277 K using a Bruker BVT30 control unit. Data acquisition and processing were performed with Bruker TOPSPIN software (Bruker Biospin GmbH, Germany).

1D ^1^H HRMAS NMR spectra were recorded at a spinning speed of 6 kHz using a zgpr pulse sequence (from Bruker's standard pulse program library) with water suppression. The ^1^H HRMAS NMR spectra were acquired using a spectral width of 8000 Hz, domain data points 16 k, number of averages 512 with 8 dummy scans, constant receiver gain of 2048, an acquisition time of 170 ms, and a relaxation delay of 2 s. All spectra were processed by an exponential window function corresponding to a line broadening of 1 Hz and zero-filled before Fourier transformation. ^1^H HRMAS NMR spectra were phased manually and automatically baseline corrected using TOPSPIN 4.0.6 (Bruker Biospin GmbH, Germany). The total analysis time (including sample preparation, optimization of NMR parameters, and data acquisition) of ^1^H HRMAS NMR spectroscopy for each sample was about 15 min.

2D homo-nuclear correlation spectroscopy (^1^H-^1^H COSY) was performed, at a spinning speed of 6 kHz, in magnitude mode using Bruker’s standard pulse program library. The parameters used for COSY were 2048 data points collected in the t2 domain over the spectral width of 4 k, 512 t1 increments were collected with 16 transients, relaxation delay 2 s, acquisition time 116 ms, and pre-saturated water resonance during relaxation delay. Prior to Fourier Transformation, the resulting data were zero filled with 2048 data points and were weighted with sine bell window functions in both dimensions.

### HRMAS NMR data analysis and quantification

All the 1D spectra acquired were referenced, calibrated, baseline, and phase corrected and analyzed by using TOPSPIN 3.1 (Bruker Biospin GmbH, Germany). Multivariate analyses of the one-dimensional NMR spectra were performed as described earlier^43^. The analysis was performed by using the SIMCA software package (Version 14.0, Umetrics, Umeå, Sweden). Bucket tables were generated from the one-dimensional spectra of control and PET NPs treated embryos and the larger water signal was excluded in the region between 4.80 and 6.00 ppm. The one-dimensional spectra were normalized to the total intensity and binned into buckets of 0.04 ppm, using MestReNova v.12.0.4. The differences in the overall metabolite concentration between individual samples were mean-centered and scaled to unit variance, and then normalized based on the Pareto method in the SIMCA software package (Version 14.0, Umetrics, Umeå, Sweden). Partial least-squares discriminant analysis (PLS-DA) was performed on the data using the SIMCA software as described earlier^43^. The model validation and significance were determined from the R2 value (that indicate how well the model fits the data), and Q2 value (that is a measure of how well the model predicts new data).

The metabolites were quantified by using Chenomx NMR Suite 8.2 (Chenomx Inc., Edmonton, Alberta, Canada). This enabled qualitative and quantitative analysis of an NMR spectrum by fitting spectral signatures from an HMDB database to the spectrum. The 600 MHz library from Chenomx was utilized which uses the concentration of a known reference signal (in our case TSP) to determine the concentration of individual compounds. The concentrations of metabolites were calculated according to a ratio relative to tCr as previously described^44^. Statistical analysis of NMR quantification was performed by one-way analysis of variance (ANOVA) using OriginPro v.8 (OriginLab, Northampton, MA, USA). Tukey test was used for multiple comparisons. A *p*-value of < 0.05 was considered significant. To check the false discovery rate, the *p*-values were corrected for multiple testing, and q-values were obtained using the Benjamini–Hochberg method^76^. Homogeneity of variance calculated based on Levene test (*p*-value greater than 0.05 were considered to have equal variance between groups).

## Supplementary Information

Supplementary Information.

## Data Availability

All data generated or analyzed during this study are included in this published article (and its Supplementary Information files). Additional raw data files can be available from the corresponding author on request.
