# Supplementary material for: A mechanistic understanding of the effects of polyethylene terephthalate nanoplastics in the zebrafish (Danio rerio) embryo
Source: Sci Rep. 2023 Feb 2;13:1891. doi: 10.1038/s41598-023-28712-y (PMC9894871; doi:10.1038/s41598-023-28712-y)
Supplement: Supplementary file 1 — Supplementary Information. [file 41598_2023_28712_MOESM1_ESM.docx]

**Supplementary Materials**

A mechanistic understanding of the effects of polyethylene terephthalate nanoplastics in the zebrafish (*Danio rerio*) embryo

Narmin Bashirova^1, 2^, David Poppitz^3^, Nils Klüver^4^, Stefan Scholz^4^, Joerg Matysik^2^ and A. Alia^1, 5^*

^1^Institute for Medical Physics and Biophysics, University of Leipzig, Leipzig, Germany

^2^Institute for Analytical Chemistry, University of Leipzig, Leipzig, Germany

^3^Institute of Chemical Technology, University of Leipzig, Leipzig, Germany

^4^Dept. Bioanalytical Ecotoxicology, Helmholtz Centre for Environmental Research-UFZ, Leipzig, Germany

^5^Leiden Institute of Chemistry, Leiden University, Leiden, The Netherlands


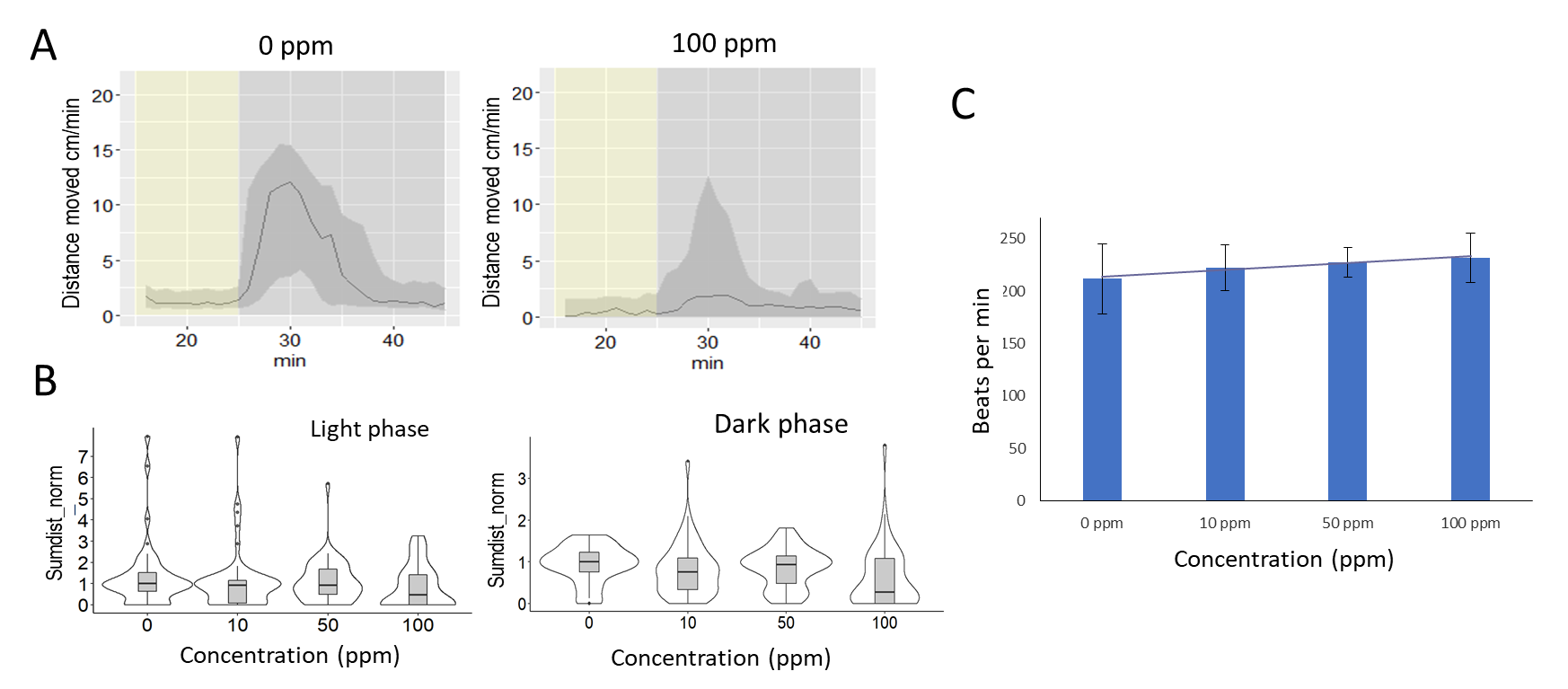


**Fig. S1**: The effect of PET NPs on the locomotor activity and heart beat frequency of zebrafish embryos at 96 hpf after exposure to PET NPs for 72 h. (A) Mean distance moved per 1min time bin (dark line with 25^th^ and 75^th^ quantile) in unexposed (0 ppm) and PET NPs (100 ppm) exposed group. Light phase (15-25min, yellow shaded) and dark phase (25-45 min, light grey). Activity response of zebrafish embryos for each treatment groups are based on a sample size of n=24. (B) Violin plot shows the normalized sum distance in the light and dark phase after exposure to various concentrations of PET NPs (0, 10, 50 or 100 ppm). (C) Heart rate analysis in beats per min.


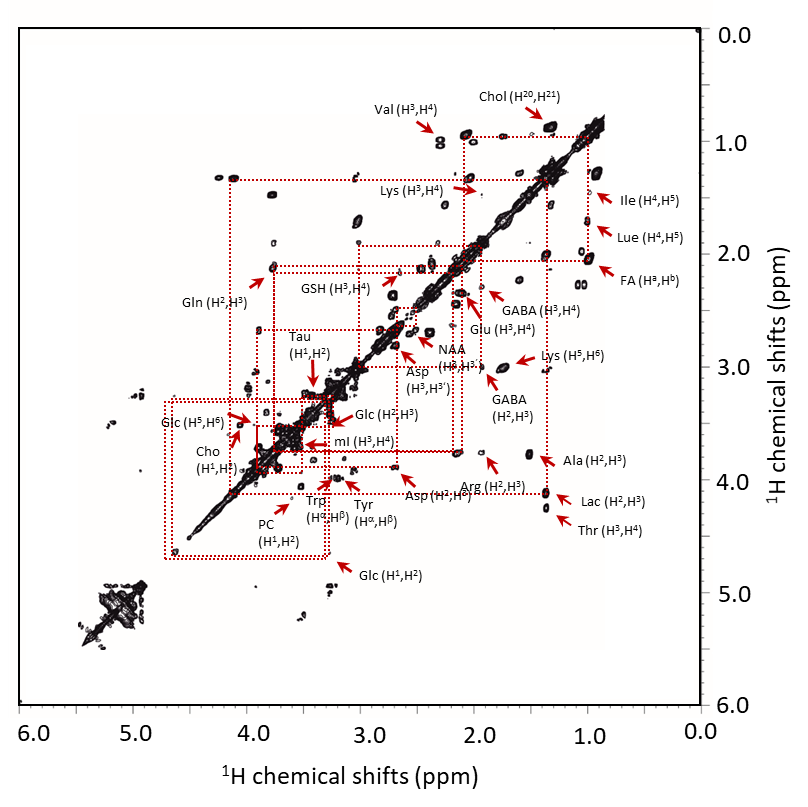


**Fig. S2.** ^1^H-^1^H COSY spectra of 96 hpf embryo in the range of 0 to 6 ppm. Measurement of ^1^H-^1^H COSY spectra and assignment of the cross peaks is made according to ref [35]. The parameters used for COSY were 2048 data points collected in the t2 domain over the spectral width of 9k, 512 t1 increments were collected with 16 transients, relaxation delay 2 sec, acquisition time 114 msec, and pre-saturated water resonance during relaxation delay. The resulting data were zero filled with 512 data points and were weighted with the squared sine bell window functions in both dimensions prior to Fourier Transformation. Application of gradient pulses along with tradition ^1^H-^1^H COSY sequence provides highly resolved spectrum.

**Table S1.** Relative changes in metabolites of zebrafish embryos exposed to PET compared to controls. Embryos exposed to 100 ppm PET at 72 hpf (for 24h), and concentration of metabolites (relative to total Cr), measured by HRMAS NMR compared to controls. For statistically significant changes, *p*-values are given; “n.s.” indicates differences are not significant. For abbreviations, see Figure 6 legend.

| **Metabolite** |  | **%Change^1^** | ***p*-value** | |  |  |
| --- | --- | --- | --- | --- | --- | --- |
|  |  |  | |  |  |  |
| Phe |  | +17.7 | | < 0.05 |  |  |
| Trp |  | +47.9 | | <0.001 |  |  |
| Tyr |  | +24.0 | | < 0.01 |  |  |
| Leu |  | -16.2 | | < 0.05 |  |  |
| Ile |  | -25.1 | | < 0.01 |  |  |
| Val |  | -18.9 | | < 0.05 |  |  |
| Glu |  | -21.0 | | < 0.05 |  |  |
| Gln |  | -22.9 | | < 0.01 |  |  |
| Gly |  | -35.6 | | < 0.01 |  |  |
| Ala |  | -24.5 | | < 0.05 |  |  |
| Cys |  | -26.7 | | < 0.05 |  |  |
| GABA |  | -22.09 | | n.s. |  |  |
| GSH |  | -33.2 | | < 0.01 |  |  |
|  |  |  | | | |  |
| Glc |  | -16.06 | | < 0.05 |  |  |
| Lac |  | +37.8 | | < 0.05 |  |  |
| ATP |  | -19.5 | | < 0.05 |  |  |
| NADH |  | -23.4 | | < 0.05 |  |  |
| Acetate |  | +11.8 | | < 0.05 |  |  |
|  |  |  | |  |  |  |
| Chol |  | +24.7 | | < 0.05 |  |  |
| FA |  | +56.3 | | < 0.01 |  |  |
| m-Ins |  | +28.5 | | < 0.05 |  |  |
| Cho |  | +104.6 | | <0.001 |  |  |
| GPC |  | +34.2 | | < 0.01 |  |  |
| EA |  | +37.08 | | < 0.05 |  |  |

^1^Percent increase (+) or decrease (−) for metabolite for treated (100 ppm PET NPs) compared to control

**Table S2.** Relative change in branched chain (BCAA) and aromatic (AAA) amino acids measured by HRMAS NMR in zebrafish embryos exposed to PET NPs (100 ppm) versus controls. Given are mean concentrations (mm, normalized to total creatine), and standard deviation (SD), as well percent change (%Change) in metabolite (relative to control). Significant difference indicated by calculated *p*-values from ANOVA.

|  |  | **Concentration** | | | | | | | | | | |  |  |
| --- | --- | --- | --- | --- | --- | --- | --- | --- | --- | --- | --- | --- | --- | --- |
|  | **Metabolite^a^** | **Control ± SD** | | | |  | **PET NPs ± SD** | | | | | **%Change** | | ***p*-Value** |
|  |  |  |  | |  |  |  | |  | |  | |  |  |
| **AAA** | | | | | |  |  |  | |  | | |  |  |
|  | Phe | 0.27 | ± | | 0.02 |  | 0.35 | | ± | | 0.04 | | 117.7% | <0.05 |
|  | Tyr | 0.42 | ± | | 0.03 |  | 0.57 | | ± | | 0.04 | | 124.0% | <0.05 |
|  | Trp | 0.28 | ± | | 0.04 |  | 0.42 | | ± | | 0.05 | | 147.9% | <0.001 |
|  |  |  |  | |  |  |  | |  | |  | |  |  |
| **BCAA** | |  | |  | |  |  |  | |  | | |  |  |
|  | Leu | 1.09 | ± | | 0.04 |  | 0.78 | | ± | | 0.09 | | 83.7% | <0.05 |
|  | Ile | 0.72 | ± | | 0.05 |  | 0.54 | | ± | | 0.11 | | 74.8% | <0.05 |
|  | Val | 0.54 | ± | | 0.05 |  | 0.36 | | ± | | 0.05 | | 81.0% | <0.05 |
|  |  |  |  | |  |  |  | |  | |  | |  |  |
| **AAA/BCAA** | | 0.41 | ± | | 0.6 |  | 0.79 | | ± | | 0.5 | | 51.8% | <0.05 |
|  | |  |  | |  |  |  | |  | |  | |  |  |
